# Complexity of Inheritance of Pathogenic Mutations Associated with Epilepsy in Consanguine Families from Pakistan

**DOI:** 10.3390/genes17020157

**Published:** 2026-01-29

**Authors:** Khajista Tahira, Anwar Ullah, Fazl Ullah, Jeena Aziz, Muhammad Ishaq Javed, Aasma Kiyani, Azra Khanum, Kerstin Hallmann, Tobias Baumgartner, Rainer Surges, Pakeeza Arzoo Shaiq, Wolfram S. Kunz

**Affiliations:** 1 Department of Epileptology, University Bonn Medical Center, 53127 Bonn, Germany; khajista04@gmail.com (K.T.); tobias.baumgartner@ukbonn.de (T.B.);; 2 University Institute of Biochemistry and Biotechnology, PMAS Arid Agriculture University Rawalpindi, Rawalpindi 46000, Pakistan; ishaq.redo01@gmail.com (M.I.J.); azrakhanum@uaar.edu.pk (A.K.); 3 Institute of Paramedical Sciences, Khyber Medical University Peshawar, Peshawar 25100, Pakistan; anwarbch.ipms@kmu.edu.pk (A.U.); khanjeena890@gmail.com (J.A.); 4 District Headquarters (DHQ) Hospital Alpuri Shangla, Alpuri 19100, Pakistan; fazlullahkmc46@gmail.com; 5 National Institute of Rehabilitation Medicine, Islamabad 44000, Pakistan; dr.kiyani.psych@gmail.com; 6 Institute of Experimental Epileptology and Cognition Research, University Bonn, 53127 Bonn, Germany

**Keywords:** recessive epilepsy genes, consanguine families, inheritance of pathogenic mutations

## Abstract

**Background/Objectives**: Consanguine families are helpful to identify recessive candidate genes for inherited diseases, but can also show an unusual inheritance pattern of pathogenic mutations. In this case series, we demonstrate this in five consanguine families with epilepsy from Pakistan. **Methods**: We performed whole exome sequencing of respective index patients, analyzed the data using two different models for inheritance of mutations and determined the segregation pattern of relevant mutations in the families by bi-directional Sanger sequencing. **Results**: Apart from mutations in classical dominant epilepsy genes (*TSC2*, *DEPDC5*, and *CACNA1I*), pathogenic mutations in rare recessive epilepsy-related genes (*PGAP2*, *NOVA2*, and *CCDC88C*) were also identified. Interestingly, we were able to provide evidence that *GALR2* is potentially an additional gene associated with a recessive form of epilepsy. In one family, a homozygous ‘pathogenic’ *TRAF3IP1* p. Gly387* nonsense mutation was identified, which, most probably due to stop-codon read-through, did not contribute to the phenotype. **Conclusions**: Our case series of consanguine families with epilepsy exemplifies the inheritance pattern of mutations in rare recessive epilepsy genes, and shows that mutations in classical epilepsy genes showing dominant or sporadic inheritance can also be relevant. That requires the analysis of whole exome data on the basis of different inheritance models.

## 1. Introduction

Epilepsy is the most frequent neurological disorder with a lifetime risk of ~3%, and genetic factors are known to be a frequent underlying cause of many epilepsies [1]. Exome based, short-read, next-generation sequencing (WES) has led to a strong acceleration in gene discovery, and there are now more than a thousand genes associated with epilepsy [2]. Recent advances, mainly due to improved exome coverage, have improved the diagnostic yield of genetic sequencing in the epilepsies, in particular for developmental and epileptic encephalopathies where around 50% of patients receive a genetic diagnosis through testing by WES [3,4]. However, despite substantial progress, especially for sporadic pediatric cases which undergo trio-exome sequencing, the majority of individuals with epilepsy who undergo sequencing do not receive a genetic diagnosis, particularly for more common epilepsy types [5].

The most frequently detected mutations associated with focal epilepsies are located in genes belonging to the MTOR pathway (*DEPDC5* [6,7], *TSC1* [8], *TSC2* [9], *NPRL2* [10] and *NPRL3* [10]), while in patients with generalized epilepsies, frequently neuronal ion channel gene mutations (sodium [11], potassium [12], calcium [13] channels and GABA receptors [14]) are observed. All these frequently epilepsy-associated genes show a dominant inheritance pattern. Recessive epilepsy-associated mutations have been less frequently reported and are therefore potentially underdiagnosed. They are probably playing a more frequent role for the epilepsy phenotype in consanguine families. To evaluate the role of recessive mutation under conditions of consanguinity, we studied in the present report the segregation pattern of epilepsy-associated mutations in five consanguine families originating from Pakistan. We observed a complex pattern of inheritance of pathogenic mutations which has important consequences for genetic diagnostics and counseling.

## 2. Materials and Methods

### 2.1. Human Subjects and DNA Collection

Genomic DNA from blood was isolated by routine techniques [15] or by the QIAamp^®^ DNA mini kit (Qiagen, Hilden, Germany) according to the protocol provided by the manufacturer.

### 2.2. Exome Sequencing

Whole exome sequencing (WES) was carried out as previously described [16], resulting in a 79-fold mean coverage (30-fold coverage for 88%, and 10-fold coverage for 97% of target sequences, respectively). Reads were mapped and variants were annotated as described. Filtering and variant prioritization were performed using the VARBANK data-base and analysis tool [17] at the Cologne Center for Genomics. In particular, we filtered for high-quality (coverage > 15-fold, phred-scaled quality > 25) and rare variants (MAF ≤ 0.001 based on dbSNP build 135; the 1000 Genomes database build 20110521; and the public Exome Variant Server, NHLBI Exome Sequencing Project, Seattle, build ESP6500) with predicted effects on protein sequence or splicing. To exclude pipeline-related artifacts (MAF ≤ 0.01), we filtered against variants from in-house WES datasets from 511 epilepsy patients. Verification of variants and testing of family members were performed by direct sequencing of purified PCR products performed by a commercial service. The used primers are shown in Supplementary Table S1.

### 2.3. Variant Analysis

The VARBANK built-in analysis tools allowed variant filtering by a recessive consanguine inheritance model which allowed the detection of relevant homozygous variants. These variants were prioritized on the basis of their CADD scores and their presence in GnomAD, ClinVar and OMIM databases.

Additionally, the variants from the respective index patients were filtered by a standard dominant model and prioritized on the basis of their frequency in GnomAD (<3 times), CADD scores (>25) and the presence of respective genes in a collection of 776 known dominant inherited genes associated with epilepsy.

The classification of the variants was performed according to the specific standard terminology—“pathogenic,” “likely pathogenic,” “uncertain significance,” “likely benign,” and “benign”—recommended by the ACMG [18] to describe variants identified in genes that cause Mendelian disorders. For described variants, the ClinVar classification was used.

## 3. Results

### 3.1. Case Reports

Clinical phenotyping of the presented five families from Pakistan was limited in some cases due to history taking only, due to poor access to prolonged EEG recordings and to high-resolution brain MRI.

#### 3.1.1. Family 1

**Figure 1:**
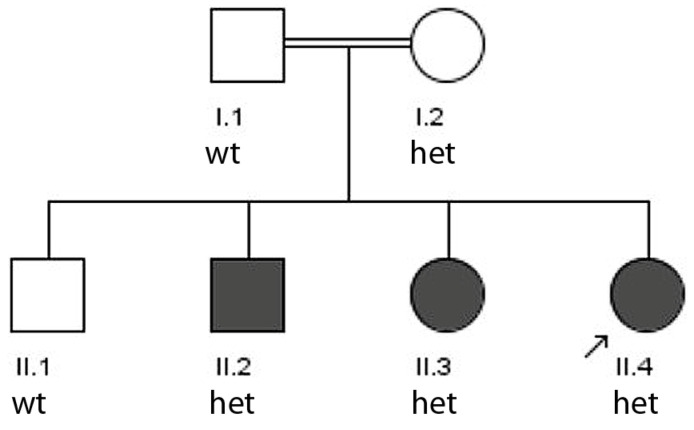
Pedigree of family 1. From all individuals, DNA samples were available. II.4 is the index patient which was whole exome sequenced (arrow). The segregation pattern of the *TSC2* mutation is indicated (wt—wild type, het—heterozygous).

The consanguine family 1 from Balochistan (the parents claimed consanguinity at the clinical interview) has three affected siblings with epilepsy (Figure 1). The male patient II.2 showed epilepsy onset at the age of 4 years. He showed tonic–clonic seizures lasting 3–4 min and had at an examination at 14 years, an abnormal EEG recording showing sharp slow waves parieto-temporooccipital. Secondary to epilepsy, he has cognitive impairment and was not able to speak. The continuous seizures were not controlled by the medication of 500 mg of sodium valproate. Additionally, he has disabled hands. The female sibling II.3 also cannot speak but was able to understand. She suffers from seizures that occur every 5–6 days.

The female index patient (II.4) had a similar disease onset (4 years), an abnormal EEG and a seizure duration of 3–4 min. The seizures were not controlled with 500 mg sodium valproate and 200 mg of carbamazepine, and she was also not able to speak but walks well. A short overview of phenotypes is given in Table 1.

Patient II.4 was whole exome sequenced. The sum of runs of homozygosity (ROH) gave a value of 30 which is inconsistent with the claimed consanguinity of parents. Accordingly, the recessive consanguine model did not present any resulting potentially pathogenic mutation. In the re-analysis of data with a dominant model, the heterozygous *TSC2* mutation p.His597Tyr (reported in ClinVar as likely pathogenic [19]) was identified, which segregated with the disease (ACMG criterion PP1), with exception of the unaffected mother, who was not affected but was also positive for the mutation. This is consistent with the reported incomplete penetrance of *TSC2* mutations being associated with familiar focal epilepsy [7].

#### 3.1.2. Family 2

**Figure 2:**
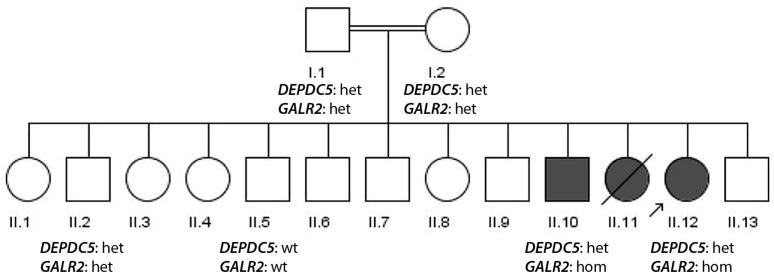
Pedigree of family 2. DNA samples were available from I.1, I.2, II.2, II.5, II.10 and II.12. The index patient is II.12 (arrow). The genotypes of the two mutations are indicated (wt—wild type, het—heterozygous, hom—homozygous).

The consanguine family 2 has three affected siblings with epilepsy (the female patient II.11 was deceased at age of 3 years due to high fever and seizures) and originates from Kohistan (KPK). Its pedigree is depicted in Figure 2.

The male patient (II.10) underwent the clinical examinations at 14 years old. He had a disease onset at 3 years old and has an abnormal EEG. He is non-syndromic but intellectually disabled and had a paresis of his left arm. The female index patient (II.12) underwent clinical examination at 11 years old. She is suffering from attention deficit hyperactivity disorder and had an onset of epilepsy at the age of 5 years. She had at examination, presented a normal EEG, and is non-syndromic. She experiences tonic–clonic seizures. The patients received medication doses of 25 mg topiramate and 1 mg risperidone. An overview of the phenotypes of the patients is presented in Table 2.

Patient II.12 was whole exome sequenced. The ROH sum value was 246 which is consistent with the consanguinity of parents. The data were therefore analyzed with a recessive consanguine inheritance model which scored a rare homozygous *GALR2* splice acceptor mutation c.369-5C>A very high. We tested the presence of this mutation in the available DNA samples and observed it in patient II.10, also in the homozygous state, while individual II.2 was heterozygous, II.5 wild type and both parents heterozygous confirming co-segregation (VUS, ACMG criteria PM2, PP1). A reanalysis of exome sequencing data from patient II.11 with a dominant inheritance model additionally revealed the rare *DEPDC5* mutation p. Thr1145Ile (VUS, ACMG criteria PM2, PP3). Segregation analysis of this mutation revealed that all individuals apart from II.5 harbored the mutation in the heterozygous state. This is not in disagreement with dominant inheritance, but the very low penetrance (33%, while usual pathogenic *DEPDC5* mutations have around 65% penetrance [20]) makes a considerable phenotype contribution quite unlikely.

#### 3.1.3. Family 3

**Figure 3:**
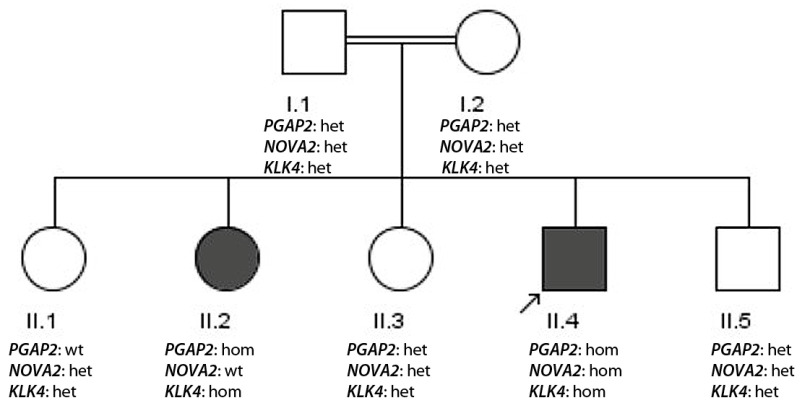
Pedigree of family 3. DNA samples were available from all individuals. The index patient is II.4 (arrow). The genotypes of the three mutations are indicated (wt—wild type, het—heterozygous, hom—homozygous).

The consanguine family 3 with two affected siblings with epilepsy (Figure 3) originates from Islamabad. The female patient (II.2) underwent the clinical examination at 15 years old. She is non-syndromic with a disease onset at 8 years. She has drug-resistant tonic–clonic seizures and was receiving a combination of 1500 mg sodium valproate, 600 mg carbamazepine and 50 mg topiramate. Her EEG showed generalized epileptic discharges. Her brother (II.4) is the index patient and underwent the clinical examination at 13 years old. He is syndromic showing intellectual disability with an epilepsy onset at the age of 9 years. He cannot speak but walks properly. He suffers from frequent tonic–clonic seizures under a combination of 1500 mg sodium valproate, 600 mg carbamazepine and 50 mg topiramate. An overview of the phenotypes of the patients is presented in Table 3.

Whole exome sequencing of patient II.4 revealed a ROH sum value of 408 which is consistent with severe consanguinity of parents. The exome data were analyzed with a recessive consanguine model and the following homozygous mutations were observed: *PGAP2*: p.Arg238Pro, reported in ClinVar as pathogenic/likely pathogenic [21] associated with Cerebellar ataxia, intellectual disability, and disequilibrium syndrome 3; *KLK4*: p.Leu211fs; c.632delT, reported in ClinVar as pathogenic [22] and associated with Amelogenesis imperfecta type 2A1; *NOVA2*: c.-1 C>A (start score change); VUS not reported in ClinVar (ACMG criteria PM2, PP3), but recessive mutations have been associated with a neurodevelopmental disorder with or without autistic features and/or structural brain abnormalities. Segregation analysis indicated that only the index patient and the affected individual II.2 harbored the homozygous pathogenic *PGAP2* and *KLK4* mutations, while II.2 was wild type for the *NOVA2* mutation. That shows that in severe consanguinity, more than one pathogenic mutation can be detected. Re-analysis of data with a dominant inheritance model revealed no relevant epilepsy-associated mutations.

#### 3.1.4. Family 4

**Figure 4:**
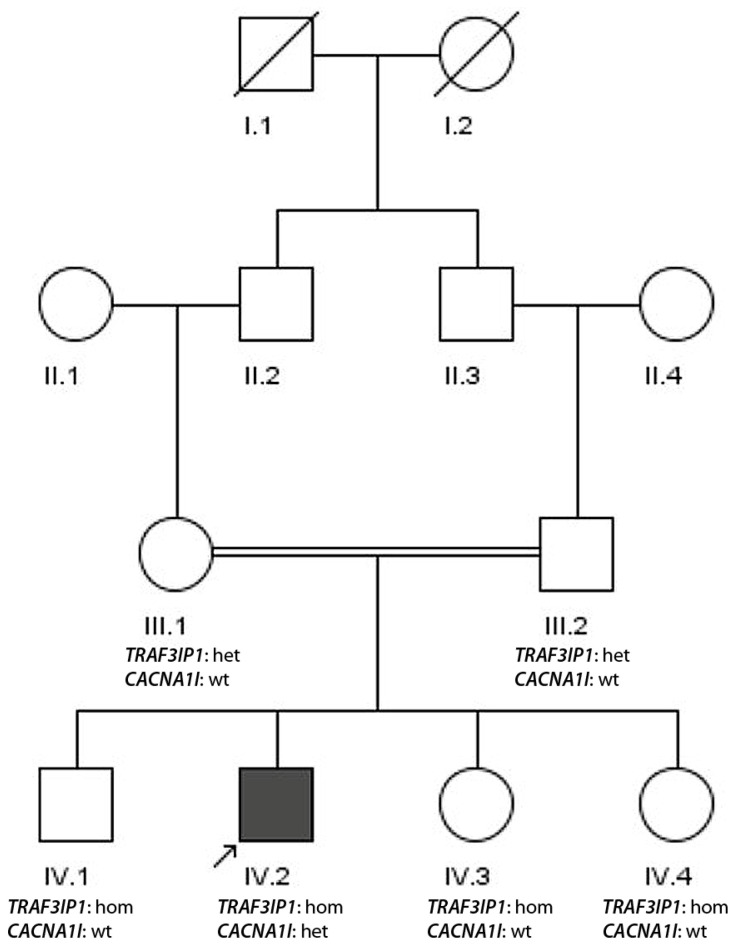
Pedigree of family 4. The index patient (IV.2) is indicated by the arrow. From individuals III.1, III.2, IV.1, IV.2, IV.3 and IV.4, DNA samples were available. The genotypes of the two mutations are indicated (wt—wild type, het—heterozygous, hom—homozygous).

The consanguine family 4 with one affected sibling with epilepsy (Figure 4) originates from the Kohat region of Khyber Pakhtunkhwa, Pakistan. The male index patient (IV.2) suffered from seizures. At the clinical examination at the age of 22 years, an abnormal EEG and a normal MRI of the brain were assessed, which is consistent with the clinical diagnosis of generalized epilepsy. The renal functional parameters (blood creatinine—0.7 mg/dL (normal range 0.6–1.1 mg/dL) and serum sodium, potassium and chloride composition) were in the normal range. Under treatment with sodium valproate (serum level 93.2 ng/mL (therapeutic range 50–100 ng/mL)), sufficient control of seizures was achieved.

Exome sequencing of the index patient revealed a ROH sum of 218 consistent with consanguinity. The analysis of data by the recessive consanguine model resulted in the homozygous nonsense mutation in *TRAF3IP1*; p. Gly387*. This mutation has been reported in ClinVar as pathogenic [23] and associated with Senior-Loken syndrome (a severe ciliopathy resulting in kidney disease) (ACMG criteria PVS1, PP3).

Since there was no clinical evidence for Senior-Loken syndrome, we performed a re-analysis of data with the dominant model and observed a *CACNA1I*; p. Tyr1524Cys mutation, described in GnomAD once as low-frequency variant with a CADD score of 32.0. Segregation analysis of mutations showed heterozygosity for the *TRAF3IP1*; p. Gly387* mutation in the parents (III.1 and III.2), but homozygosity in all children (IV.1, IV.2, IV.3 and IV.4) excluding any relevant contribution to the phenotype. In contrast, all family members, apart from the patient IV.2, were wild type for the *CACNA1I*; p.Tyr1524Cys mutation, confirming a de novo appearance (ACMG criteria PS2, PP3) which would be consistent with the generalized epilepsy of this individual.

#### 3.1.5. Family 5

**Figure 5:**
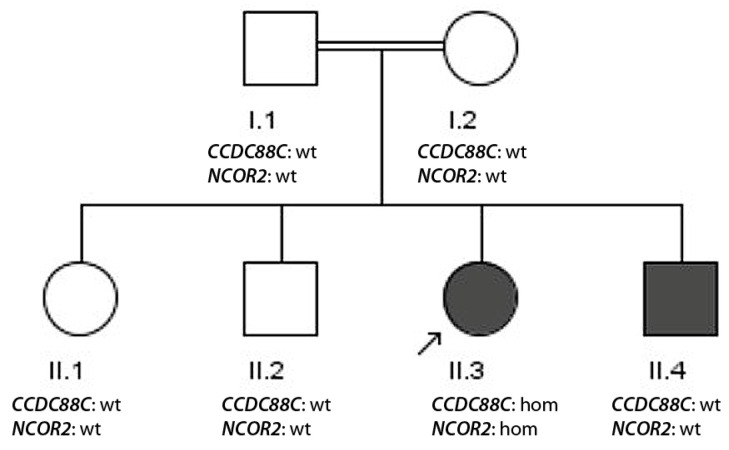
Pedigree of family 5. DNA samples were available from all individuals. The index patient is II.3 (arrow). The genotypes of the two mutations are indicated (wt—wild type, hom—homozygous).

The consanguine family 5 with two affected siblings with epilepsy (Figure 5) originates from Gujrat (Punjab). Its pedigree is shown in Figure 5. The 24-year-old index patient (II.3) suffered from focal seizures. A disease onset at the age of 1 year was reported, and in the early examinations, vascular abnormalities in the brain were detected. The patient has—apart from epilepsy which could not be controlled by the used ASM (500 mg sodium valproate and 200 mg of levetiracetam)—behavioral abnormalities, no proper speech and intellectual disability. EEG and MRI were also abnormal. Her male sibling (II.4) underwent clinical examination at 20 years old and had an abnormal EEG. His seizures could be controlled with 500 mg of sodium valproate. Additionally, the patient suffered from intellectual disability and had deformation of hands and feet, as well as a disease onset at the age of 2 years. An overview about the phenotypes of the patients is presented in Table 4.

Exome sequencing of the index patient revealed a ROH sum of 307 consistent with consanguinity. Analysis of data with a recessive consanguine model identified the homozygous *CCDC88C* nonsense mutation p.Arg536Glufs*13; c.1606delA not described in ClinVar. Since this mutation results in a knock-out of this gene, this mutation can be considered to be potentially pathogenic for patient II.3 (ACMG criteria PVS1, PM2, PP3). In OMIM, recessive nonsense mutations in *CCDC88C* have been associated with congenital hydrocephalus, which could explain the vascular brain abnormalities detected in the index patient. Segregation analysis of this mutation within the family revealed a major problem that all other family members were wild type for this mutation (and also for another homozygous mutation detected in *NCOR2,* p.Asp552Tyr). Since a resampling of DNA from all family members did not eliminate this issue, a baby mix-up in early childhood appears a very likely reason for the segregation discrepancy.

## 4. Discussion

Here, we present the results of segregation analysis of potentially pathogenic mutations in five consanguine families with epilepsy. In our case series we observed the presence of the following constellations:

(i) Dominant inheritance of a known epilepsy-associated mutation (family 1, *TSC2*).
(ii) Simultaneous presence of a recessive variant and a dominant variant in epilepsy genes segregating with disease (family 2, *GALR2* and *DEPDC5*).
(iii) Multiple presence of recessive variants segregating with disease (family 3, *PGAP2*, *KLK4* and *NOVA2*) in highly consanguine families.
(iv) De novo appearance of a pathogenic epilepsy-associated mutation in a consanguine family (family 4, *CACNA1I*) in the additional presence of a homozygous pathogenic nonsense mutation in *TRAF3IP1* not co-segregating with disease.
(v) Missing segregation due to sample mismatch, which might generate problems in genetic counseling (family 5, *CCDC88C*).

Thus, the presented constellations illustrate problems associated with the identification of the real epilepsy-associated mutations.

Constellation (i) represents a typical finding for families with inherited focal epilepsy and confirms the observed *TSC2* variant p.His597Tyr [19] as clearly pathogenic (classified so far by three submissions as likely pathogenic).

Constellation (ii) shows co-segregation of potentially two different epilepsy-related genes: the frequent epilepsy gene, *DEPDC5* [5] and a putative novel gene, *GALR2*, which has been shown in various reports to be a valuable target for antiepileptic drugs [24]. The *DEPDC5* variant p.Thr1145Ile is very rare (absent in GnomAD) and located at a quite conserved site of the genes (as indicated by the relatively high Polyphen-2 score of 0.981). However, the segregation analysis indicated that many of unaffected family members harbored the mutation leading to a disease penetrance of only 33%, while pathogenic *DEPDC5* usually has a penetrance around 65% [20]. That makes a sole contribution of this variant to the quite severe phenotype of the reported patients—severe epilepsy with early disease onset and intellectual disability or hyperactivity—unlikely. Therefore, we hypothesize that the homozygous c.369-5C>A splice acceptor mutation in *GALR2* is a more likely candidate to explain the severe phenotype of the patients.

Constellation (iii) is an expected problem for genetic diagnostics in severe consanguine families. Here, several pathogenic mutations in relevant recessive genes can potentially contribute to the phenotype of patients: *PGAP2*: p.Arg238Pro, likely pathogenic [21] (Cerebellar ataxia, intellectual disability, and disequilibrium syndrome 3) and *NOVA2*: c.-1 C>A (start score change). Additionally, a homozygous *KLK4*: p.Leu211fs; c.632delT; pathogenic [22] (Amelogenesis imperfecta type 2A1) was detected. Since only the mutations in *PGAP2* and *KLK4* segregated with the disease, they potentially are contributing both to the phenotype of the patients.

The constellation (iv) for family 4 is a quite unexpected finding. While the de novo *CACNA1I* mutation would sufficiently explain the generalized epilepsy in the index patient, the obviously missing effect of the homozygous nonsense mutation c.1159G>T; p.Gly387* in *TRAF3IP1* on the phenotype of the patient and its three healthy siblings deserves some additional explanation. In silico examination of this mutation resulted in a CADD value of 49.0 and an additional a splice site effect (Pangolin-0.650; the last nucleotide of the exon is mutated). On the basis of these data, this variant has been classified as pathogenic (ACMG criteria PVS1, PP3) in ClinVar [23]. Biallelic pathogenic variants in *TRAF3IP1* are associated with ciliopathies usually presenting with a strong renal phenotype [25,26]. However, the detailed analysis of this variant shows that it is a GGA>UGA mutation resulting in a UGA stop codon. Very importantly, UGA read-through has been frequently described [27], in particular in the synthesis of selenoproteins, where it is context-dependent, associated with incorporation of selenocysteine [28].

Constellation (v) indicates a problem which might be very relevant for genetic counseling of patients. If a laboratory-related sample mix-up can be excluded (by independent repeated sampling), the genetic segregation workup is able to identify issues, like a potential baby mix-up. Interestingly, in our constellation, the index patient harbored the homozygous *CCDC88C* nonsense mutation p.Arg536Glufs*13; c.1606delA. Recessive loss-of-function mutations have been associated with congenital hydrocephalus [29], which could explain the detected vascular abnormalities in the brain of patient II.3 from family 5.

Our study has certain limitations. They include the small sample size of five families, limitations in clinical phenotyping, the lack of replication, and the limited statistical inference. Therefore, the presented findings are hypothesis-generating, and not representative. Moreover, more complex inheritance models might allow a more detailed analysis of the presented genetic data.

## 5. Conclusions

Our report illustrates several genetic constellations in consanguine families with epilepsy which can be relevant for appropriate genetic diagnostics and counseling. In families with multiple rare variants, there is a risk of misattributing causality. A two-step analysis of whole exome data at least by a recessive and a dominant model is required, and in constellations with multiple rare variants, an additional segregation analysis of mutations is recommended to identify the most likely disease-associated mutations.

## Figures and Tables

**Table 1:** Overview of phenotypes of patients from family 1.

Patient	Seizure Type	Age of Onset	EEG Findings	Developmental Outcome
II.2	tonic–clonic	4 years	abnormal	cognitive impairment
II.3	tonic–clonic	5 years	abnormal	not able to speak
II.4	tonic–clonic	4 years	abnormal	not able to speak

**Table 2:** Overview of phenotypes of patients from family 2.

Patient	Seizure Type	Age of Onset	EEG Findings	Developmental Outcome
II.10	tonic–clonic	3 years	abnormal	intellectual disability
II.11	not known	<3 years	not known	deceased
II.12	tonic–clonic	5 years	normal	hyperactive

**Table 3:** Overview of phenotypes of patients from family 3.

Patient	Seizure Type	Age of Onset	EEG Findings	Developmental Outcome
II.2	tonic–clonic	8 years	abnormal	non-syndromic
II.4	tonic–clonic	9 years	abnormal	intellectual disability

**Table 4:** Overview of phenotypes of patients from family 5.

Patient	Seizure Type	Age of Onset	EEG Findings	Developmental Outcome
II.2	tonic–clonic	1 year	abnormal	intellectual disability
II.4	tonic–clonic	2 years	abnormal	intellectual disability

## Data Availability

The exome sequencing data presented in this study are available on request from the corresponding author due to ethical reasons.

## Supplementary Materials

The following supporting information can be downloaded at https://www.mdpi.com/article/10.3390/genes17020157/s1, Table S1: Primer sequences used in this study.

## Abbreviations

The following abbreviations are used in this manuscript:

ASM	Anti-seizure medication
MRI	Magnetic resonance imaging
ROH	Run of homozygosity
