# Supplementary material for: Complexity of Inheritance of Pathogenic Mutations Associated with Epilepsy in Consanguine Families from Pakistan
Source: Genes (Basel). 2026 Jan 29;17(2):157. doi: 10.3390/genes17020157 (PMC12940928; doi:10.3390/genes17020157)
Supplement: Supplementary file 1 [file genes-17-00157-s001.zip › genes-4098653-supplementary.pdf]

Supplementary Table S1. Primers sequences used in this study

Family	gene	sequence
Family 1	TSC2Ex.17F	GCTGCGTCCTCTCTCTGCA
Family 1	TSCEx.17R	CTGCAGCCGGATGCTGCTC
Family 2	GALREx.2F	CATGCGCTAAGGACCTTCCTC
Family 2	GALREx.2R	ACCAGCACAGGAAGCAGGTAG
Family 2	DEPDC5Ex.35F	TGCATACGTGCCATCTTGCTT
Family 2	DEPDC5Ex.35R	CTGGTGAGAAGTACAGTCATG
Family 3	PGAP2Ex.6F	TAGTCGTCATTCTTGCTGCC
Family 3	PGAP2Ex.6R	TGGATCCTATCCTGGCCTCA
Family 3	NOVA2x1F	AGCCGCCGCAGCAGCAGCAG
Family 3	NOVA2x1R,1	agccgcagccctttctcac
Family 3	NOVA2x1R,2	ttctcacCTCCCGTGTTGCTG
Family 3	KLK4Ex.6F	GCAGGATTAGATGAAGTCAGG
Family 3	KLK4Ex.6R	TGCAGAGGTTGGTGTAGACAC
Family 4	CACNA1Ix27F	taccaactcgaccagatacac
Family 4	CACNA1Ix27R	TCAGAGCTATAAGCAGCAGCT
Family 4	TRAF3IP1x8F	GAATCATGTGGAGGCTGAGA
Family 4	TRAF3IP1x8R	ATCAGCCACACCACTGTGAG
Family 5	CCD88CEx.14F	AGCAGAGCAACCAAGATCTG
Family 5	CCD88CEx.14R	GCTCTCCGTCTTGCAGGCAG
Family 5	NCOR2Ex.17F	GCAGCCAGTGCAAATCCAGC
Family 5	NCOR2Ex.17R	TGATGGCCTCCTCGCTGTTG
